# Development of a triplex RT-qPCR assay for simultaneous quantification of Japanese encephalitis, Murray Valley encephalitis, and West Nile viruses for environmental surveillance

**DOI:** 10.1128/spectrum.01364-24

**Published:** 2024-08-20

**Authors:** Yawen Liu, Wendy J Smith, Metasebia Gebrewold, Stuart L. Simpson, Xinhong Wang, Warish Ahmed

**Affiliations:** 1 State Key Laboratory of Marine Environmental Science, College of the Environment & Ecology, Xiamen University, Xiamen, China; 2 CSIRO Environment, Ecosciences Precinct, Dutton Park, Queensland, Australia; Institute of Microbiology, Beijing, China

**Keywords:** wastewater, JEV, MVEV, WNV, triplex-RT-qPCR, surveillance

## Abstract

The co-circulation of mosquito-borne Japanese encephalitis virus (JEV), Murray Valley encephalitis virus (MVEV), and West Nile virus (WNV) has impacted human and animal health in multiple countries worldwide. To facilitate early warnings and surveillance of the presence of these viral infectious agents in the environment, a triplex reverse transcription-quantitative PCR (RT-qPCR) was developed for simultaneous quantification of JEV, MVEV, and WNV in potential hotspots such as piggery and urban wastewater and environmental water samples. The performance of the developed triplex RT-qPCR assay was compared with that of simplex counterparts, all using the same primer and probe sequences. The quantifiable results showed a concordance rate of 93.9%–100% (Cohen’s kappa) between the triplex and simplex assays. The mean concentrations of exogenous JEV, MVEV, and WNV using the triplex and simplex RT-qPCR assays were remarkably similar in piggery/urban wastewater and environmental water samples. However, the impacts of the matrix effects (i.e., sample composition and PCR inhibition) of environmental water samples on the accurate quantification of these viruses need to be considered. Taken together, this newly developed triplex RT-qPCR assay of JEV, MVEV, and WNV will allow for a more rapid and cost-efficient sample analysis and data interpretation. The application of the triplex assay for environmental surveillance may be a valuable tool to complement the existing disease and mosquito surveillance approaches used to safeguard the health of both humans and animals.

**IMPORTANCE:**

The co-circulation of mosquito-borne Japanese encephalitis virus (JEV), Murray Valley encephalitis virus (MVEV), and West Nile virus (WNV) poses significant threats to human and animal health globally. In this study, a triplex RT-qPCR assay was developed for simultaneous quantification of these viruses in wastewater and environmental water samples. Results demonstrated high concordance and sensitivity of the newly developed triplex RT-qPCR assay compared to simplex assays, indicating its efficacy for environmental surveillance. This cost-effective and rapid assay offers a vital tool for timely monitoring of mosquito-borne viruses in environmental samples, enhancing our ability to mitigate potential outbreaks and safeguard public health.

## INTRODUCTION

Japanese encephalitis virus (JEV) in the genus *Flavivirus* within the family *Flaviviridae* is responsible for severe Japanese encephalitis outbreaks in Asia and western Pacific countries and is of growing concern for spread from northern to southern Australia (1). It is estimated that approximately 68,000 Japanese encephalitis cases have occurred annually in 24 countries (1). For example, the recent unprecedented emergence of JEV was reported across a wide geographical area of southeastern Australia in 2022, affecting 70 piggeries across four states and resulting in 30 confirmed human infections, including five deaths (2). JEV is a mosquito-borne virus with a complex transmission ecology that involves ardeid wading birds and *Culex*. mosquitoes in an enzootic transmission cycle (2). Pigs serve as the primary amplifying hosts, leading to epizootic spillover to humans, with higher risks encountered at piggeries (3). Murray Valley encephalitis virus (MVEV) and West Nile virus (WNV), both within the JEV serological complex, share the same zoonotic transmission cycles with JEV and co-circulate in multiple global regions (4). WNV is the most widely distributed virus among encephalitis *flaviviruses*, with significant outbreaks recorded across Asia, Africa, Europe, North America, and Australia (5). MVEV is the most important cause of arboviral neurological disease in humans in Australia, causing the case fatality rates to be 15% to 30% and with 30% to 50% of patients suffering from long-term neurological sequelae (6–8). Despite the disease burden with their infections, few vaccines and therapies are currently available (9, 10). The transmission of JEV, MVEV, and WNV is projected to increase due to factors such as climate change and increased interactions at the humans–livestock–wildlife interface (11, 12).

To mitigate the growing impacts of their global transmission and emergence in new territories, disease surveillance strategies in humans and animals as well as mosquito-based surveillance strategies are currently deployed (13, 14). Although disease surveillance remains the main pillar for JEV, MVEV, and WNV monitoring, a large portion of human and animal infections are asymptomatic, with estimations showing that only 1 in 50 to 1 to 1,000 human infections for these three viruses exhibit clinical symptoms (13–15). This scenario would lead to a significant number of unreported cases in disease surveillance reports (12). The reliance on mosquito surveillance, which involves capturing mosquitoes as vectors to estimate the prevalence of viruses in vector populations, has challenges with respect to its validity as accurate proxies for assessment of true mosquito-borne virus exposure risk in human populations (16).

Environmental surveillance, on the other hand, bypasses the influence and biases associated with human behavior and health systems by looking into pathogens present in the community-level sewerage systems. Data from environmental sections (e.g., wastewater) offers a comprehensive view of infection burden, including both symptomatic and asymptomatic individuals. Detecting viruses in wastewater enables earlier outbreak identification compared to traditional methods, as viral shedding can precede the onset of clinical symptoms in humans and animals. Additionally, wastewater surveillance offers a population-level perspective by collecting data from a community rather than individuals. This approach allows for the detection of outbreaks even when clinical symptoms are not apparent or when only a small proportion of the population is sampled through traditional surveillance methods (17–19). The feasibility of wastewater surveillance in monitoring mosquito-borne viruses such as Zika and dengue viruses has already been demonstrated (20, 21). A case study of JEV detection in municipal wastewater during a disease outbreak also highlights the potential of wastewater surveillance as a complementary layer to overcome the constraints of mosquito-/sentinel-based surveillance (22). Beyond human settings, animal agriculture in proximity interactions with wildlife and human habitats had been one of the driving forces in accelerating the emergence of mosquito-borne diseases (23–25). Oral/fecal shedding of JEV (26–28), MVEV (29), and WNV (30) from infected humans and animals may precede the onset of clinical symptoms, facilitating early detection and prompt intervention of potential population-/community-level outbreaks (25, 29, 31–33).

For environmental surveillance, the reverse transcription quantitative polymerase chain reaction (RT-qPCR) has been widely adopted due to its high sensitivity and ability to generate quantitative results (34, 35). Despite its effectiveness, the use of RT-qPCR is limited by cost and throughput (i.e., generally one target is analyzed at a time) (36). These limitations become particularly apparent during instances of co-circulation of JEV, MVEV, and WNV, stemming from the shared ecological and geographical distribution of amplifying hosts and vectors (37–39). To allow for a rapid and sensitive quantification of JEV, MVEV, and WNV, a triplex RT-qPCR assay would offer an advantage in the simultaneous quantification of these viral targets in clinical and as well as environmental samples (36, 40).

In this study, a triplex RT-qPCR assay using TaqMan™ probes was developed and validated to allow rapid, sensitive, and cost-effective monitoring of JEV, MVEV, and WNV in environmental samples. The newly developed triplex assay underwent rigorous validation against simplex assays. The performance of this assay was further assessed by quantifying exogenous JEV, MVEV, and WNV in wastewater and environmental water samples. As a high-efficiency tool, triplex RT-qPCR assay would significantly enhance the effectiveness of environmental surveillance as a part of early warning, facilitating timely intervention to manage the circulation of JEV, MVEV, and WNV at the human–animal–environmental interface.

## MATERIALS AND METHODS

### RT-qPCR standards

Gamma-irradiated (50 kGy) JEV (NSW/22), MVEV (OR156), and WNV (2311–01-1506) control materials were provided by Australian Centre for Disease Preparedness (ACDP) laboratory, CSIRO. RNA was extracted from JEV, MVEV, and WNV control materials using the QIAamp Viral RNA Mini Kit (Qiagen, Cat. No. 52904, Dusseldorf, Germany) based on the manufacturer’s instructions. RNA was eluted with 100 µL of buffer AVE. The concentrations (copy numbers) of JEV, MVEV, and WNV in extracted RNA samples from control materials were determined using reverse transcription digital PCR (RT-dPCR) assays (Table S1). JEV, MVEV, and WNV standard curves (1 × 10^5^ to 1 copies/μL of RNA) were prepared from known concentrations of the respective RNA samples.

### Optimization of JEV, MVEV, and WNV simplex RT-qPCR assays

For the development of the JEV, MVEV, and WNV triplex RT-qPCR assay, previously published RT-qPCR assays that had been widely adopted for clinical and mosquito surveillance were used (41, 42). The sequences of primers and probes for each assay are provided in Table 1. Simplex RT-qPCR assays were optimized separately through primer/probe titration and gradient PCR to test for the optimal annealing temperature for each virus (Table S2). Simplex RT-qPCR amplifications were performed in 20-µL reaction mixtures using 5 µL of TaqMan™ Fast Virus 1-Step Master Mix (Applied Biosystem, California, USA) and 2 µL of control RNA (1 × 10^4^ copies/μL for each virus). The threshold and baseline for each simplex assay were adjusted to the same value above the background fluorescence and within the phase of exponential amplification (JEV: 100 RFU; MVEV: 100 RFU; WNV: 62 RFU). The optimized simplex RT-qPCR assay conditions (primer and probe concentrations and annealing temperatures) are presented in Table 1.

**TABLE 1:** Optimized simplex and triplex JEV, WNV, and MVEV RT-qPCR assays used this study

Targets (references)	Assay types	Primers and probes (5’–3’)	Primer and probe concentrations	Cycling parameters
JEV (41)	Simplex	F: GCC ACC CAG GAG GTC CTT	400 nM	10 minutes at 50°C, 45 cycles of 15 seconds at 95°C, and 60 seconds at 56°C
		R: CCC CAA AAC CGC AGG AAT	400 nM	
		P: FAM-CAA GAG GTG GAC GGC C-BHQ1	400 nM	
MVEV (42)	Simplex	F: ATY TGG TGY GGA AGY CTC A	700 nM	10 minutes at 50°C, 45 cycles of 15 seconds at 95°C, and 60 seconds at 57.9°C
		R: MGC RTA GAT GTT YTC AGC CC	700 nM	
		P: FAM-ATG TYG CYC TGG TCC TGG TCC CT-BHQ1	600 nM	
WNV (42)	Simplex	F: AAC CCC AGT GGA GAA GTG GA	400 nM	10 minutes at 50°C, 45 cycles of 15 seconds at 95°C, and 60 seconds at 56°C
		R: TCA GGC TGC CAC ACC AAA	400 nM	
		P: FAM-CGA TGT TCC ATA CTC TGG CAA ACG-BHQ1	400 nM	
JEV, MVEV, and WNV (41, 42)	Triplex	F:(JEV): GCC ACC CAG GAG GTC CTT	300 nM	10 minutes at 50°C, 45 cycles of 15 seconds at 95°C, and 60 seconds at 62°C
		R:(JEV): CCC CAA AAC CGC AGG AAT	300 nM	
		P(JEV): FAM-CAA GAG GTG GAC GGC C-BHQ1	100 nM	
		F(MVEV): ATY TGG TGY GGA AGY CTC A	500 nM	
		R(MVEV): MGC RTA GAT GTT YTC AGC CC	500 nM	
		P(MVEV): Texas red-ATG TYG CYC TGG TCC TGG TCC CT-BHQ2	100 nM	
		F(WNV): AAC CCC AGT GGA GAA GTG GA	100 nM	
		R(WNV): TCA GGC TGC CAC ACC AAA	100 nM	
		P(WNV): Cy5-CGA TGT TCC ATA CTC TGG CAA ACG-BHQ2	100 nM	

### Optimization of triplex RT-qPCR assay

To examine the discrepancies (if any) of Cq values obtained between simplex and triplex assays for each target, initially duplex RT-qPCR assays of (i) JEV and WNV, (ii) JEV and MVEV, and (iii) WNV and MVEV were set up using the same primer and probe concentrations and cycling parameters as used in the optimized simplex RT-qPCR assays. Duplex and simplex RT-qPCR amplifications were performed in parallel with a 20 µL reaction volume containing 5 µL of TaqMan™ Fast Virus 1-Step Master Mix and 2 µL of control RNA (1 × 10^4^ copies/μL of JEV, MVEV, and WNV) for each assay. No discrepancy of Cq values was observed between the (JEV and WNV and JEV and MVEV) duplex assays and corresponding simplex counterparts. However, the WNV and MVEV duplex RT-qPCR assay showed discrepancy in Cq values (Table S3), and therefore, were both further optimized by adjusting the primer and probe concentrations for each target separately. Optimized WNV and MVEV duplex RT-qPCR assay were then used for the development of triplex RT-qPCR assay. Finally, the triplex RT-qPCR assay was tested in a gradient PCR to determine the optimal annealing temperature. The optimal primer and probe concentrations and cycling parameters for optimized triplex RT-qPCR assay are listed in Table 1.

Tenfold serial dilutions (1 × 10^5^ to 1 copies/μL) prepared from JEV, MVEV, and WNV RNA (i.e., quantified using dRT-qPCR) were used to evaluate the amplification curves using the all three optimized simplex and triplex RT-qPCR assays (Fig. S1). To ascertain potential competitions among JEV, MVEV, and WNV primers/probes in the triplex assay mix, an orthogonal experimental design was employed, incorporating various combinations of RNA concentrations (Table 2). Triplex RT-qPCR amplifications were performed with a 20-µL reaction mixture containing 5 µL of TaqMan™ Fast Virus 1-Step Master Mix, 300 nM of each primer (JEV), 500 nM of each primer (MVEV), 100 nM of each primer (WNV), and 100 nM of the probe (JEV, MVEV, and WNV). All simplex and triplex RT-qPCR assays were performed in quadruplicate using a Bio-Rad CFX96 thermal cycler equipped with six different channels. For each RT-qPCR run, quadruplicate non-template controls were included.

**TABLE 2:** Orthogonal experimental design to determine the competition among JEV, MVEV, and WNV in the triplex RT-qPCR assay^*a*^

Copies/reaction (viruses)	Cq (Mean ± SD)		
	JEV	MVEV	WNV
2 × 10^4^ (JEV) +2 × 10^1^ (MVEV) +2 × 10^1^ (WNV)	20.5 ± 0.1	ND	32.2 ± 0.8
2 × 10^3^ (JEV) +2 × 10^2^ (MVEV) +2 × 10^2^ (WNV)	23.8 ± 0.1	28.3 ± 0.1	28.5 ± 0.3
2 × 10^2^ (JEV) +2 × 10^3^ (MVEV) +2 × 10^3^ (WNV)	27.2 ± 0.1	24.6 ± 0.2	25.1 ± 0.3
2 × 10^1^ (JEV) +2 × 10^4^ (MVEV) +2 × 10^4^ (WNV)	30.6 ± 0.1	21.1 ± 0.2	21.1 ± 0.1
2 × 10^4^ (JEV) +2 × 10^4^ (MVEV) +2 × 10^1^ (WNV)	20.5 ± 0.1	21.3 ± 0.1	32.8 ± 1.3
2 × 10^3^ (JEV) +2 × 10^3^ (MVEV) +2 × 10^2^ (WNV)	23.8 ± 0.1	24.8 ± 0.1	28.7 ± 0.1
2 × 10^2^ (JEV) +2 × 10^2^ (MVEV) +2 × 10^3^ (WNV)	27.4 ± 0.1	28.0 ± 0.2	25.3 ± 0.1
2 × 10^1^ (JEV) +2 × 10^1^ (MVEV) +2 × 10^4^ (WNV)	30.6 ± 0.1	31.9 ± 0.6	21.1 ± 0.3
2 × 10^4^ (JEV) +2 × 10^1^ (MVEV) +2 × 10^4^ (WNV)	20.5 ± 0.1	ND	21.2 ± 0.1
2 × 10^3^ (JEV) +2 × 10^2^ (MVEV) +2 × 10^3^ (WNV)	23.9 ± 0.1	28.4 ± 0.1	25.3 ± 0.2
2 × 10^2^ (JEV) +2 × 10^3^ (MVEV) +2 × 10^2^ (WNV)	27.3 ± 0.1	24.7 ± 0.1	28.6 ± 0.1
2 × 10^1^ (JEV) +2 × 10^4^ (MVEV) +2 × 10^1^ (WNV)	30.8 ± 0.2	21.2 ± 0.1	32.3 ± 0.1
2 × 10^4^ (JEV) +2 × 10^4^ (MVEV) +2 × 10^4^ (WNV)	20.4 ± 0.1	21.1 ± 0.1	21.2 ± 0.1
2 × 10^3^ (JEV) +2 × 10^3^ (MVEV) +2 × 10^3^ (WNV)	23.9 ± 0.1	24.7 ± 0.1	25.5 ± 0.1
2 × 10^2^ (JEV) +2 × 10^2^ (MVEV) +2 × 10^2^ (WNV)	27.2 ± 0.1	27.9 ± 0.2	29.1 ± 0.2
2 × 10^1^ (JEV) +2 × 10^1^ (MVEV) +2 × 10^1^ (WNV)	30.5 ± 0.2	31.6 ± 0.3	32.4 ± 0.5

^*a*^ ND: not detected; SD: standard deviation.

### Seeding experiments

To validate the efficacy of the newly developed triplex RT-qPCR assay in comparison with the optimized simplex assays for quantification of JEV, MVEV, and WNV, archived environmental water (i.e., ponds within a piggery), piggery wastewater, and untreated urban wastewater samples were seeded with varying quantities of gamma-irradiated JEV, MVEV, and WNV. Environmental water samples were collected from the Wide Bay in QLD, Australia. Piggery wastewater samples were collected from a piggery lagoon located in a pig farm in the outskirts of Brisbane, Australia. Untreated urban wastewater samples were collected from a wastewater treatment plant (WWTP) located in QLD, Australia. The WWTP receives wastewater from a catchment with approximately 151,000 people. From here on, all these environmental samples will be referred to as “water/wastewater” samples. All samples were collected using a sterile PET bottle and then stored at 4°C. Before seeding, all environmental samples were confirmed negative for JEV, MVEV, and WNV using the optimized simplex RT-qPCR assays. For each water/wastewater type, three to four individual samples were combined to create composite water/wastewater samples, each measuring approximately 1.5 to 2 L. After thorough manual mixing, 15 mL of piggery wastewater, 50 mL of untreated urban wastewater, and 100 mL of pond water were aliquoted from corresponding composite samples and then transferred into Falcon tubes (15–50 mL, Eppendorf, Hamburg, Germany). The different sample volumes were chosen based on the water/wastewater turbidity and considering dilution effects of viral targets in these samples.

Prior to the exogenous virus seeding, different titer of JEV, MVEV, and WNV were prepared from serial dilutions of control materials. Subsequently, 11 working control materials containing varying quantities of JEV, MVEV, and WNV were prepared. An aliquot of 10 µL of each control material was seeded into individual water samples for each water/wastewater type. A total of 12 samples were prepared for each water/wastewater type, including a method negative control sample, which was seeded with 10 µL of nuclease-free water.

### Water/wastewater sample concentration and extraction

Exogenous JEV, MVEV, and WNV were concentrated from the water/wastewater samples using the modified adsorption–extraction (AE) workflow. This workflow had been demonstrated to yield better recovery of RNA viruses compared to other workflows in a method comparison study (43). Individual water/wastewater samples were filtered through MF-Millipore 0.45-µm MCE membranes (47 mm; Cat no. HAWP04700) (Millipore, Burlington, Massachusetts, USA) via the filter flask (Merck Millipore Ltd.). After filtration, the membrane was rolled and transferred into a 5-mL bead-beating tube (Qiagen, Hilden, Germany) for RNA extraction.

A Qiagen RNeasy PowerWater Kit (Cat. No. 14700–50-NF) was used to extract nucleic acids from the membranes. Briefly, the membrane in the 5-mL bead-beating tube was lysed with 850 µL of buffer PM1 (contains guanidine hydrochloride), 150 µL of TRIzol reagent (Ambion, Sigma-Aldrich, California), and 10 µL of 2-mercaptoethanol (Gibco, Waltham, USA). The bead-beating tubes containing lysed samples were homogenized with a Precellys 24 homogenizer (Bertin Technologies, Montigny-le-Bretonneux, France). Homogenization parameters were set at a speed of 9,000 rpm for 15 seconds per cycle. Three cycles were performed, each separated by 10-s intervals. Following homogenization, the tubes were centrifugated at 4,000 *g* for 4 minutes to separate the filter debris and beads from the supernatant. After centrifugation, each sample lysate supernatant was transferred into a 2-mL tube and added with 200 µL of IRS solution provided with the Qiagen RNeasy PowerWater Kit. The 2-mL tubes were incubated at 4°C for 10 minutes and then centrifugated at 13,000 *g* for 1 minute. The supernatant was transferred to a rotor adapter on the QIAcube Connect platform and processed following the manufacturer’s protocol. The RNA sample was then eluted with 100 µL of nuclease-free water and stored at −20°C prior to simplex and triplex RT-qPCR analyses. All simplex and triplex RT-qPCR assays were performed on the same day to mitigate potential RNA degradation during the freeze–thaw cycle.

### Quality assurance and quality control

The amplification efficiencies (E), correlation coefficient (*r*^2^), and y-intercepts were derived from the standard curves, assay limit of detection (ALOD), and PCR inhibition provided based on the Minimum Information of Publication of Quantitative Real-Time PCR Experiments (MIQE) guidelines for the optimized simplex and triplex assays (44). The assay limit of detection (ALOD) was defined as the minimum copy number with a 95% probability of detection for simplex and triplex RT-qPCR assays (45).

A murine hepatitis virus (MHV) real-time RT-PCR assay was applied to determine PCR inhibition in extracted water/wastewater RNA samples by adding known copy number (10^4^) of MHV RNA (46). The reference quantification cycle (Cq) value was compared with the Cq values obtained from all water/wastewater RNA samples. Water/wastewater samples were considered to have no PCR inhibition when the Cq values of environmental RNA samples were within two Cq values of the reference Cq value (47). The RT-qPCR set up for all assays and sample concentration/extraction were performed in separate laboratories to minimize contamination introduced in experiments. The sample negative control and the concentration and extraction negative control were included in processing of all water/wastewater samples to account for any contamination during the seeding and extraction experiments. Gamma-irradiated virus seeding into wastewater as well as wastewater sample concentration and extraction were conducted in a Biosecurity Containment Level 2 (BC2) laboratory and within a biosafety cabinet to minimize exposure to potential pathogens present in wastewater samples.

### Data analysis

Environmental water/wastewater samples seeded with known concentrations of viruses were categorized as being positive for each virus if amplification was observed within 45 cycles in a minimum of one out of three RT-qPCR replicates. Samples were categorized as non-detectable if no amplification was observed in any of the RT-qPCR replicates. Samples were considered quantifiable if amplifications were detected in at least two out of three RT-qPCR replicates, and the Cq values were above the ALOD for each target (48). The concentrations of quantifiable water/wastewater samples were log_10_-transformed and expressed as log_10_ copies/50 mL.

All data were tested for normality and homogeneity of variances before being subject to statistical analysis. Cohen’s kappa (κ) was computed to gage the level of agreement with 95% confidence intervals (CIs) between the quantification of three viruses using the simplex and newly developed triplex RT-qPCR assays. The paired *t*-test and correlation analysis were employed to evaluate statistical differences and correlation coefficients in the concentrations of each target obtained between the simplex and triplex RT-qPCR assays. For correlation analysis, the results across all piggery wastewater, untreated urban wastewater, and environmental water samples were combined, including non-quantifiable and non-detected (substituted by half of ALOD) samples (48). In addition, the concentration results for each target obtained by the simplex and triplex RT-qPCR assays were fitted into a linear regression model to calculate the slopes of the least squares line of best fit. Goodness of fit was calculated to evaluate the performance of each model. The above mentioned analysis was performed using GraphPad Prism Version 8.3.1 (GraphPad Software, La Jolla, CA, USA). Statistical tests were considered significant if *P* < 0.05.

## RESULTS

### RT-qPCR performance characteristics

For both optimized simplex and triplex RT-qPCR assays, the correlation coefficients (*r*^2^) of RT-qPCR standard curves (1 × 10^5^ to 1 copy/μL) for JEV, MVEV, and WNV were within the range between 0.990 and 0.998. The Y-intercepts of RT-qPCR standard curves were 34.90 and 34.61 for simplex JEV and triplex JEV, 35.55 and 35.88 for simplex MVEV and triplex MVEV, and 35.31 and 36.85 for simplex WNV and triplex WNV, respectively. The RT-qPCR efficiencies were 100% and 102% for simplex JEV and triplex JEV, 98.1% and 96.5% for simplex MVEV and triplex MVEV, and 103% and 91.5% for simplex WNV and triplex WNV (Table S4), which were within the prescribed MIQE guidelines (44). The ALODs for simplex JEV, MVEV, and WNV assays were 1.0, 1.9, and 7.6 copies/reaction, while the ALODs for triplex JEV, MVEV, and WNV assays were 1.2, 6.9, and 8.8 copies/reaction, respectively. The ALODs for the triplex RT-qPCR assays were comparable to simplex counterparts, falling within the same order of magnitude. All water/wastewater RNA samples were within the 2 Cq values of the reference Cq value, suggesting the absence of PCR inhibitors in all RNA samples.

### Competition among three targets in the triplex RT-qPCR assay

Serial dilutions (2 × 10^4^ to 2 × 10^1^ copies/reaction) of standards extracted from control materials of three viruses were utilized to assess the competition among three targets in the triplex RT-qPCR. The Cq values of all three targets were similar when the standard concentrations were the same in RT-qPCR reactions. When the concentrations of three standards were paired with each other orthogonally, JEV and WNV amplifications were not affected in the presence of high concentrations of other targets. However, in the presence of 2 × 10^4^ copies of JEV and/or WNV, no amplification was observed for 2 × 10^1^ copies of MVEV.

### Performance of triplex RT-qPCR assays for quantification of three viruses

Across the 33 water/wastewater samples tested, the concordance between the optimized simplex and triplex RT-qPCR assays for JEV, MVEV, and WNV was 96.9% (kappa coefficient of 0.939 with a 95% CI of 0.822 to 1), 100% (kappa coefficient of 1 with a 95% CI of 1 to 1), and 93.9% (kappa coefficient of 0.879 with a 95% CI of 0.718 to 1), respectively. One environmental water sample was quantifiable for JEV by the simplex RT-qPCR assay, but was not quantifiable (i.e., positive) by the triplex RT-qPCR assay (Table 3). For WNV, two untreated urban wastewater samples were quantifiable by the simplex RT-qPCR assay, but was below the ALOD by the triplex assay.

**TABLE 3:** Concentrations of JEV, MVEV, and WNV in piggery wastewater, untreated urban wastewater, and environmental water samples determined using the simplex and triplex RT-qPCR assays^*b-g*^

Water/wastewater type	Sample no.	Mean ± SD log_10_ copies/50 mL^*a*^					
		Triplex JEV	Simplex JEV	Triplex MVEV	Simplex MVEV	Triplex WNV	Simplex WNV
Piggery wastewater	P1	5.64 ± 0.06	5.64 ± 0.06	5.68 ± 0.03	5.67 ± 0.12	6.27 ± 0.07	6.21 ± 0.08
	P2	4.63 ± 0.04	4.50 ± 0.05	4.63 ± 0.10	4.61 ± 0.03	5.14 ± 0.10	5.20 ± 0.03
	P3	3.00 ± 0.19	3.15 ± 0.09	3.23 ± 0.10	3.48 ± 0.24	3.94 ± 0.22	4.04 ± 0.07
	P4	NQ	NQ	ND	ND	ND	ND
	P5	ND	ND	ND	NQ	ND	ND
	P6	4.41 ± 0.07	4.40 ± 0.07	4.47 ± 0.02	4.38 ± 0.02	ND	ND
	P7	4.58 ± 0.09	4.59 ± 0.01	ND	ND	ND	ND
	P8	4.55 ± 0.06	4.66 ± 0.02	ND	ND	5.00 ± 0.19	5.22 ± 0.09
	P9	NQ	NQ	4.66 ± 0.03	4.66 ± 0.16	4.93 ± 0.21	5.19 ± 0.08
	P10	NQ	NQ	ND	ND	4.66 ± 0.24	4.90 ± 0.11
	P11	ND	ND	3.00 ± 0.05	2.81 ± 0.17	ND	ND
Urban wastewater	U1	5.34 ± 0.03	5.35 ± 0.03	5.38 ± 0.08	5.22 ± 0.10	6.06 ± 0.01	5.82 ± 0.07
	U2	4.09 ± 0.10	4.08 ± 0.07	4.21 ± 0.05	4.05 ± 0.19	4.70 ± 0.22	4.87 ± 0.36
	U3	2.73 ± 0.03	2.75 ± 0.04	3.38 ± 0.03	3.31 ± 0.04	3.68 ± 0.12	3.86 ± 0.02
	U4	NQ	NQ	NQ	NQ	ND	2.89 ± 0.37
	U5	ND	NQ	ND	ND	ND	ND
	U6	3.99 ± 0.07	3.97 ± 0.06	4.01 ± 0.01	3.93 ± 0.26	ND	ND
	U7	4.09 ± 0.07	4.06 ± 0.10	ND	ND	ND	ND
	U8	4.13 ± 0.03	4.20 ± 0.04	NQ	NQ	4.78 ± 0.04	4.50 ± 0.26
	U9	NQ	NQ	4.26 ± 0.03	3.72 ± 0.44	4.59 ± 0.13	4.41 ± 0.27
	U10	NQ	NQ	NQ	NQ	4.56 ± 0.14	4.86 ± 0.01
	U11	NQ	NQ	4.25 ± 0.02	4.23 ± 0.05	ND	2.78 ± 0.11
Environmental water (pond water)	E1	4.22 ± 0.03	4.26 ± 0.02	4.31 ± 0.02	4.36 ± 0.15	4.90 ± 0.01	4.97 ± 0.18
	E2	3.07 ± 0.02	3.27 ± 0.10	3.15 ± 0.06	3.14 ± 0.05	3.83 ± 0.10*^c^*	4.21 ± 0.04*^c^*
	E3	NQ	1.88 ± 0.06	2.24 ± 0.01	2.31 ± 0.13	2.62 ± 0.06^*d*^	3.08 ± 0.11^*d*^
	E4	ND	ND	ND	ND	NQ	NQ
	E5	ND	ND	ND	ND	ND	ND
	E6	3.08 ± 0.08	3.35 ± 0.04	3.23 ± 0.04	2.95 ± 0.58	ND	ND
	E7	3.01 ± 0.04	3.18 ± 0.05	ND	ND	NQ	NQ
	E8	2.78 ± 0.01	3.01 ± 0.09	ND	ND	3.09 ± 0.13^*e*^	3.84 ± 0.06^*e*^
	E9	ND	ND	3.08 ± 0.01^*b*^	3.22 ± 0.03^*b*^	3.08 ± 0.04f	3.81 ± 0.04f
	E10	ND	ND	NQ	NQ	3.36 ± 0.05	3.73 ± 0.02
	E11	ND	ND	3.40 ± 0.10	3.32 ± 0.11	ND	NQ

^*a*^ Concentrations were log_10_-transformed and expressed as log_10_ copies/50 mL.

^*b-g*^ Values labeled with the same letters show a statistically significant difference; SD: standard deviation; ND: not detected; NQ: not quantifiable.

Quantification results of JEV obtained from the simplex and triplex RT-qPCR assays were similar at the individual sample level. Apart from a non-quantifiable sample determined by the triplex assay, the concentrations of JEV in all water/wastewater samples ranged from 2.75 ± 0.04 to 5.64 ± 0.06 log_10_ copies/50 mL measured by the simplex JEV, whereas the concentrations using the triplex RT-qPCR ranged from 2.73 ± 0.03 to 5.64 ± 0.06 log_10_ copies/50 mL. No significant difference (*P* > 0.05) was observed between these two assays for 17 quantifiable samples by a paired *t*-test. Similarly, comparable quantification results of MVEV were observed by the simplex and triplex RT-qPCR assays. MVEV concentrations in all water/wastewater samples ranged from 2.31 ± 0.13 to 5.67 ± 0.12 log_10_ copies/50 mL and 2.24 ± 0.01 to 5.68 ± 0.03 log_10_ copies/50 mL using the simplex MVEV and triplex assays, respectively. The paired *t*-test indicated no statistically significant differences between two assays for 17 of 18 quantifiable samples, except one environmental water sample where the concentration of MVEV was significantly different in the simplex compared to triplex RT-qPCR assay (*P* < 0.05).

For WNV, the performance of the triplex RT-qPCR assay varied depending on the type of water/wastewater samples. Apart from two negative untreated urban wastewater samples determined by the triplex assay, quantifiable WNV concentrations obtained by the simplex assay (ranging from 3.86 ± 0.02 to 6.21 ± 0.08 log_10_ copies/50 mL) and triplex assay (ranging from 3.68 ± 0.12 to 6.27 ± 0.07 log_10_ copies/50 mL) did not differ significantly from each other in piggery wastewater and untreated urban wastewater samples as per paired *t*-test (*P* > 0.05), while in environmental water samples, a significant difference was observed between the two assays for five quantifiable samples (*P* < 0.05). The concentrations in these samples ranged from 3.08 ± 0.11 to 4.97 ± 0.18 log_10_ copies/50 mL, as measured by the simplex WNV, and from 2.62 ± 0.06 to 4.90 ± 0.01 log_10_ copies/50 mL, as measured by the triplex RT-qPCR assay.

### Correlation between the simplex and triplex assays

**Fig 1:**
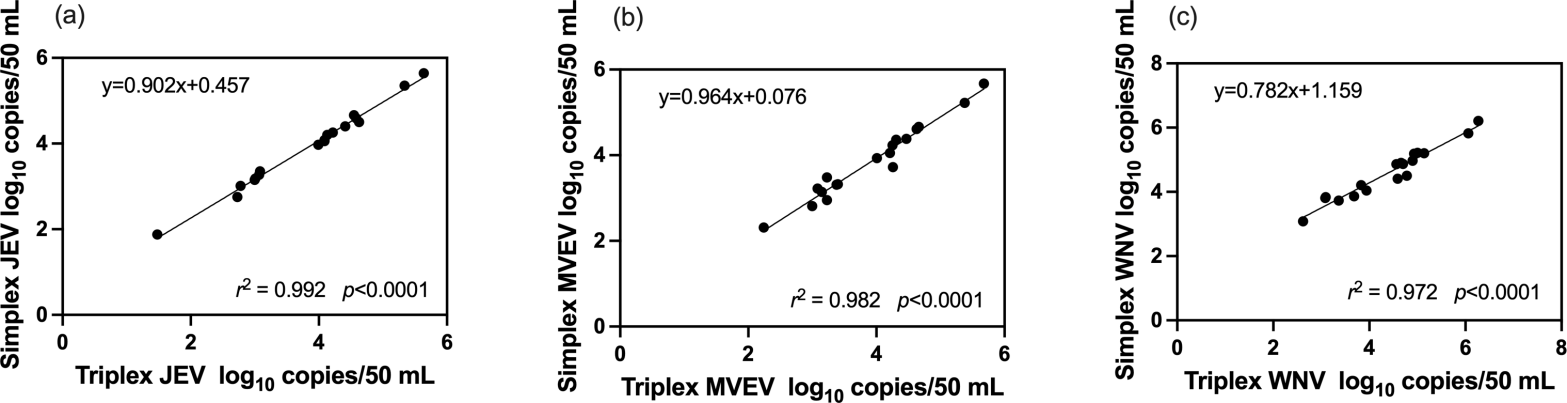
Correlations between concentrations obtained from the simplex and triplex RT-qPCR: (a) JEV, (b) MVEV, and (c) WNV using piggery wastewater, untreated urban wastewater, and environmental water samples.

Significant correlations were observed between the concentration results obtained from the simplex and triplex RT-qPCR assays, regardless of the target (*P* < 0.05), with the correlation coefficient 0.992 (95 % CI: 0.990 to 0.999) for JEV (Fig. 1a), 0.982 (95 % CI: 0.951 to 0.994) for MVEV (Fig. 1b), and 0.972 (95 % CI: 0.925 to 0.990) for WNV (Fig. 1c). In linear regression models, when the units of the X-axis were expressed as concentrations obtained from the triplex assays, the slopes of best-fit and Y-intercepts were 0.902 (95 % CI: 0.860 to 0.944) and 0.457 (95 % CI: 0.289 to 0.624) for JEV, 0.964 (95 % CI: 0.865 to 1.06) and 0.076 (95 % CI: −0.323 to 0.475) for MVEV, and 0.782 (95 % CI: 0.681 to 0.882) and 1.159 (95 % CI: 0.706 to 1.612) for WNV.

## DISCUSSION

In this study, we developed and evaluated the performance of a triplex RT-qPCR assay for simultaneous quantification of JEV, MVEV, and WNV in water/wastewater samples. The triplex RT-qPCR assay comprises three specific probes labeled with a unique fluorescent dye for each target (FAM using the blue detection channel for JEV, Texas Red using the green detection channel for MVEV, and Cy5 using the purple detection channel for WNV). There are several advantages of multiplex qPCR/RT-qPCR: (i) it allows the amplification and quantification of multiple target sequences simultaneously in a single reaction, which is more efficient in terms of time, resources, and cost compared to running three separate simplex qPCR assays; (ii) a single reaction for multiple targets conserves nucleic acid, especially when working with a small volume of samples; (iii) analyzing all targets in the same reaction reduces interassay variability since all samples are subjected to the same experimental conditions; (iv) fewer reactions decrease the chances of contamination during sample handling and processing, thereby increasing the reliability of results; (v) multiplex-qPCR simplifies data interpretation by providing simultaneous information on multiple targets, facilitating comparative analysis.

However, multiplex qPCR/RT-qPCR assays also have some limitations, including increased complexity in primer/probe design and optimization, potential cross-reactivity between primer sets compared to simplex assays. For this study, instead of designing new primer sets, we selected the most widely used, highly specific and sensitive RT-qPCR assays from research literature (41, 42). These assays have been previously used to detect/quantify JEV, MVEV, and WNV from clinical specimens and mosquito pool samples (2, 49–51). A crucial aspect of multiplex qPCR/RT-qPCR is the competition among targets for the same pool of reagents as multiple reactions would occur simultaneously in a single tube. Thus, an optimal concentration of PCR buffer, relative concentrations of primers, optimal RNA templates, and PCR conditions are essential to achieve uniform amplification of multiple targets (52).

In view of these considerations, we used single-tube format TaqMan™ Fast Virus 1-Step Multiplex Master Mix (No ROX), which is designed to enable dye flexibility for multiplexing up to four different RNA/DNA targets for high-throughput RT-qPCR. The 4X formulation enhances the detection of both RNA and DNA viral pathogens, even in the presence of challenging PCR inhibitors. We also maintained the primer concentration for each target no more than 500 nM to ensure the optimal primer-to-template ratio and facilitate the equivalent amplifications of targets with varying abundances, as previously recommended (53). In this scenario, the more abundant target will likely reach its plateau rapidly due to primer depletion, leaving sufficient reagents available for the amplification of the less abundant target (53).

For this study, varying concentrations of RNA standards were orthogonally paired with each other to evaluate the competition of three targets in triplex RT-qPCR assay. When equal concentrations of three RNA standards were used in the triplex RT-qPCR, all three tenfold standard dilutions showed linear amplifications ranging from 10^4^ to 10^1^ copies. When high (10^4^) copy numbers of MVEV and WNV were introduced in the reaction in the presence of low (10^1^) copy numbers of JEV, the Cq values of JEV did not vary significantly compared to the Cq values obtained from amplifications of all three RNA standards at equally low (10^1^) copy numbers. Similar results were also observed for MVEV. However, the amplification of MVEV in the sample with the lowest copy number (2 × 10^1^ copies/reaction) was masked in the presence of high copy numbers (2 × 10^4^ copies/reaction) of JEV or WNV. The performance characteristic parameters of all optimized simplex and triplex assays were within the MIQE guidelines (44), with the ALOD for all assays below 1 × 10^1^ copies/reaction.

Environmental surveillance has evolved into a complementary tool for providing early warning of emerging infectious diseases (17). Wastewater surveillance of mosquito-borne viruses is being currently recognized as a valuable tool to the existing disease-/mosquito-based surveillance (12, 20, 21). This approach could be further extended to livestock/aquaculture settings that serve as a sentinel for alarming potential viral disease circulation through early detection of viruses in environmental samples (25). Given that environmental samples may contain substances that inhibit the PCR and may also have interference from other components, the effectiveness of optimized single and triplex RT-qPCR assays was assessed by seeding these viruses into environmental water/wastewater samples. Seeding experiments were conducted because the presence of JEV, MVEV, and WNV in water and wastewater environments in the study area is unlikely, considering no recent outbreaks of these mosquito-borne viruses have been reported. Another reason for utilizing water/wastewater samples is the already demonstrated feasibility of monitoring JEV and WNV in urban sewerage systems (22, 54).

Furthermore, oral/fecal shedding of infectious JEV had been confirmed from infected pigs (26–28). Pigs are also susceptible to MVEV and WNV infections, as evidenced by the development of moderate to high viremia in pigs challenged with MVEV and WNV (55, 56). Taken together, the piggery wastewater and untreated urban wastewater samples hold promise as potential hotspots for detecting JEV, MVEV, and WNV. In addition, environmental water samples representing potentially mosquito breeding sites were also selected to validate the method sensitivity of the newly developed triplex RT-qPCR assay in comparison with simplex RT-qPCR assays for environmental surveillance.

The concentrations of three targets obtained from the seeded water/wastewater samples using the optimized triplex RT-qPCR assay were remarkably similar to the concentrations determined using the three optimized simplex assays. The kappa coefficients for JEV (0.939), MVEV (1), and WNV (0.879) indicated an almost perfect agreement between the triplex and simplex assays for each target. Only one environmental water sample was quantifiable for JEV (1.88 ± 0.06 log_10_ copies/50 mL), and two untreated urban wastewater samples were quantifiable for WNV (2.82 ± 0.21 log_10_ copies/50 mL) at the lowest concentration level by a simplex assay, yet non-detectable by the triplex assay. Such a discrepancy is generally observed for target concentrations that are near or at the ALOD due to sub-sampling error (57).

Based on the exogenous JEV, MVEV and WNV seeding experiments, concentrations of all three targets obtained between two assays were highly comparable for all virus-seeded piggery wastewater and urban wastewater samples. For environmental water samples, the simplex RT-qPCR appeared to be slightly more sensitive than triplex RT-qPCR in the quantification of MVEV in one sample and of WNV in five quantifiable samples. This might be attributed to specific sample matrix interferences in the wastewater, which were collected from a pond system within a piggery. During sample processing, we observed plant materials in the sample which may contain PCR inhibitors such as polyphenols and polysaccharides and have inhibited the PCR at low concentrations of targets (58, 59). Although no PCR inhibition was observed for any of the water/wastewater samples that were seeded with 10^4^ copies of MHV, a low level of inhibition is likely possible.

Collectively, the study results indicate a high detection sensitivity is achieved using the newly developed triplex RT-qPCR assay for quantification of JEV, MVEV, and WNV in environmental water, piggery wastewater, and untreated urban wastewater samples. Furthermore, this triplex assay would allow for a rapid and comparative analysis and data interpretation for environmental surveillance, with the sensitivity and accuracy supported by comparing with the optimized simplex assays. Beyond the detection of viruses in environmental water samples, the newly developed assay can also be used for detection/quantification of these three viruses in clinical and mosquito samples during their infections and disease outbreaks in a region.

## SUPPLEMENTAL MATERIAL

Supplemental material spectrum.01364-24-s0001.docx Supplemental figures and tables.

ASM does not own the copyrights to Supplemental Material that may be linked to, or accessed through, an article. The authors have granted ASM a non-exclusive, world-wide license to publish the Supplemental Material files. Please contact the corresponding author directly for reuse.
