# Supplementary material for: Development of a triplex RT-qPCR assay for simultaneous quantification of Japanese encephalitis, Murray Valley encephalitis, and West Nile viruses for environmental surveillance
Source: Microbiol Spectr. 2024 Aug 20;12(10):e01364-24. doi: 10.1128/spectrum.01364-24 (PMC11448262; doi:10.1128/spectrum.01364-24)
Supplement: Supplemental material — Supplemental figures and tables. [file spectrum.01364-24-s0001.docx]

**Development of a triplex RT-qPCR assay for simultaneously quantification of Japanese encephalitis, Murray Valley encephalitis, and West Nile Viruses**

Yawen Liu^a,b^, Wendy Smith^b^, Metasebia Gebrewold^b^, Stuart L. Simpson^b^, Xinhong Wang^a^, Warish Ahmed^b^

^a^ State Key Laboratory of Marine Environmental Science, College of the Environment & Ecology, Xiamen University, Xiamen 361102, China.

^b^ CSIRO Environment, Ecosciences Precinct, 41 Boggo Road, Dutton Park, QLD 4102, Australia

**Corresponding author.** Warish Ahmed. Mailing address: Ecosciences Precinct, 41 Boggo Road, Dutton Park 4102, Queensland, Australia. Tel.: +617 3833 5582; E-mail address: [Warish.Ahmed@csiro.au](mailto:Warish.Ahmed@csiro.au)

**Supplementary tables and figures**

**
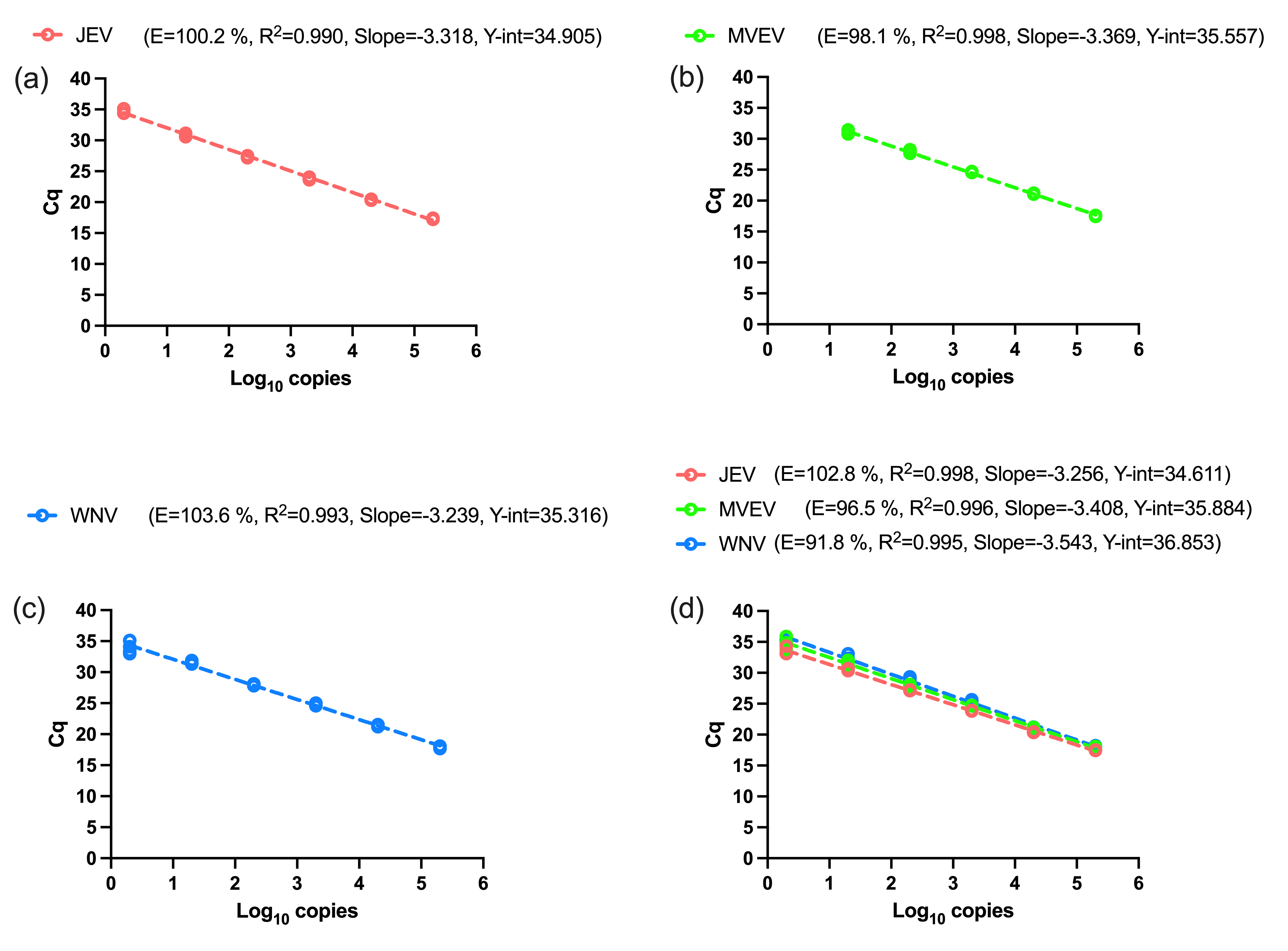
**

**Fig. S1**

**The standard curves (a) simplex JEV (b) simplex MVEV (c) simplex WNV and (d) triplex JEV, MVEV and WNV by using simple regression for the cycle threshold values versus the amount of template RNA over a range of 2 × 10^5^ to 2 copies/reaction.**

**Table ST1**

**RT-dPCR assays for JEV, WNV and MVEV used in this study.**

| Targets | Assay types | Primers and probes (5’ - 3’) | Primer and probe concentrations | Cycling parameters |
| --- | --- | --- | --- | --- |
| JEV^1^ | Simplex | F: GCC ACC CAG GAG GTC CTT | 800 nM | 10 min at 50 °C, 45 cycles of 15 s at 95 °C, 60 s at 56 °C |
|  |  | R: CCC CAA AAC CGC AGG AAT | 800 nM |  |
|  |  | P: FAM-CAA GAG GTG GAC GGC C-BHQ1 | 800 nM |  |
| MVEV^2^ | Simplex | F: ATY TGG TGY GGA AGY CTC A | 1400 nM | 10 min at 50 °C, 45 cycles of 15 s at 95 °C, 60 s at 57.9 °C |
|  |  | R: MGC RTA GAT GTT YTC AGC CC | 1400 nM |  |
|  |  | P: FAM-ATG TYG CYC TGG TCC TGG TCC CT-BHQ1 | 1200 nM |  |
| WNV^2^ | Simplex | F: AAC CCC AGT GGA GAA GTG GA | 800 nM | 10 min at 50 °C, 45 cycles of 15 s at 95 °C, 60 s at 56 °C |
|  |  | R: TCA GGC TGC CAC ACC AAA | 800 nM |  |
|  |  | P: FAM-CGA TGT TCC ATA CTC TGG CAA ACG-BHQ1 | 800 nM |  |

**Table ST2**

**Cq values of simplex RT-qPCR assays for JEV, WNV and MVEV through optimization.**

| Parameter | Concentration/temperature | Cq value (mean ± SD)^a^ | | |
| --- | --- | --- | --- | --- |
|  |  | JEV | WNV/Kunjin | MVEV |
| Primer | 300 nM | 26.08 ± 0.04 | 25.33 ± 0.22 | 26.48 ± 0.10 |
|  | 400 nM | 25.88 ± 0.14 | 25.17 ± 0.42 | 26.03 ± 0.03 |
|  | 500 nM | 25.91 ± 0.09 | 25.26 ± 0.27 | 26.06 ± 0.04 |
|  | 600 nM | 26.06 ± 0.08 | 25.38 ± 0.04 | 25.97 ± 0.12 |
|  | 700 nM | 26.18 ± 0.18 | 25.50 ± 0.29 | 25.83 ± 0.02 |
|  | 800 nM | 26.35 ± 0.14 | 26.55 ± 0.14 | 25.88 ± 0.09 |
|  | 900 nM | 26.35 ± 0.06 | 26.85 ± 0.25 | 25.88 ± 0.11 |
|  | 1000 nM | 26.42 ± 0.05 | 26.41 ± 0.36 | 25.98 ± 0.10 |
| Probe (paired with optimized primer concentration) | 100 nM (300 nM for MVEV) | 27.12 ± 0.07 | 28.29 ± 0.09 | 23.57 ± 0.14 |
|  | 200 nM (400 nM for MVEV) | 26.29 ± 0.06 | 26.79 ± 0.33 | 23.44 ± 0.10 |
|  | 300 nM (500 nM for MVEV) | 26.11 ± 0.05 | 26.42 ± 0.37 | 23.52 ± 0.01 |
|  | 400 nM (600 nM for MVEV) | 25.92 ± 0.03 | 26.47 ± 0.18 | 23.41 ± 0.04 |
|  | 500 nM (700 nM for MVEV) | 25.88 ± 0.10 | 26.34 ± 0.30 | 23.57 ± 0.03 |
| Annealing temperature (paired with optimized primer and probe concentrations) | 64.0 ℃ | 26.61 ± 0.20 | 26.41 ± 0.64 | 23.83 ± 0.10 |
|  | 63.5 ℃ | 26.37 ± 0.22 | 26.55 ± 0.22 | 23.65 ± 0.08 |
|  | 62.3 ℃ | 26.35 ± 0.16 | 26.50 ± 0.35 | 23.66 ± 0.10 |
|  | 60.4 ℃ | 26.19 ± 0.11 | 26.61 ± 0.16 | 23.56 ± 0.14 |
|  | 57.9 ℃ | 25.85 ± 0.10 | 26.81 ± 0.26 | 23.56 ± 0.07 |
|  | 56.0 ℃ | 25.72 ± 0.13 | 26.61 ± 0.10 | 23.70 ± 0.06 |
|  | 54.7 ℃ | 25.64 ± 0.19 | 27.74 ± 1.31 | 24.14 ± 0.09 |
|  | 54.0 ℃ | 25.68 ± 0.09 | 28.93 ± 0.49 | 24.61 ± 0.09 |

^a^Concentrations of primer and probe, and annealing temperature reaping the lowest Cq values were determined as the optimal for simplex assays of each target.

**Table ST3**

**Discrepancies of Cq values obtained between duplex (JEV and WNV, JEV and MVEV, MVEV and WNV) and simplex RT-qPCR assays using the same concentration of targets.**

| Assay types | JEV | | | MVEV | | | WNV | | |
| --- | --- | --- | --- | --- | --- | --- | --- | --- | --- |
|  | Cq value (mean ± SD) | | Variance^a^ | Cq value (mean ± SD) | | Variance^a^ | Cq value (mean ± SD) | | Variance^a^ |
|  | Simplex | Duplex |  | Simplex | Duplex |  | Simplex | Duplex |  |
| JEV and MVEV duplex | 21.06 ± 0.11 | 20.91 ± 0.26 | -0.15 | 26.57 ± 0.06 | 26.23 ± 0.07 | -0.34 | NA | NA | NA |
| JEV and WNV duplex | 20.68 ± 0.06 | 20.26 ± 0.04 | -0.43 | NA | NA | NA | 21.34 ± 0.15 | 21.33 ± 0.23 | -0.01 |
| WNV and MVEV duplex | ND | NA | NA | 24.13 ± 0.12 | 25.65 ± 0.40 | 1.52 | 22.22 ± 0.18 | 24.38 ± 0.81 | 2.16 |

^a^Variance was determined by means of Cq values obtained through simplex and duplex assays; NA: not applicable.

**Table ST4**

**RT-qPCR performance characteristics.**

| RT-qPCR assays | Performance characteristics | | | | |
| --- | --- | --- | --- | --- | --- |
|  | Efficiency (E) (%) | Linearity (R^2^) | Slope | Y-intercept | ALOD (copies/reaction) |
| JEV (Simplex) | 100.2 | 0.990 | -3.318 | 34.905 | 1.0 |
| MVEV (Simplex) | 98.1 | 0.998 | -3.369 | 35.557 | 1.9 |
| WNV (Simplex) | 103.6 | 0.993 | -3.239 | 35.316 | 7.6 |
| JEV (Triplex) | 102.8 | 0.998 | -3.256 | 34.611 | 1.2 |
| MVEV (Triplex) | 96.5 | 0.996 | -3.408 | 35.884 | 6.9 |
| WNV (Triplex) | 91.5 | 0.995 | -3.543 | 36.853 | 8.8 |
